# Supplementary material for: Mechanistic study on the sulfate migration in glycosaminoglycans during MS fragmentation
Source: Commun Chem. 2026 Feb 14;9:130. doi: 10.1038/s42004-026-01939-2 (PMC13018550; doi:10.1038/s42004-026-01939-2)
Supplement: Supplementary file 2 — SI PDF [file 42004_2026_1939_MOESM2_ESM.pdf]

Mechanistic Study on the Sulfate Migration in Glycosaminoglycans during MS fragmentation

Lukasz Polewski,[1,2,#] Murat Yaman,[3,4,#] Matko Tokić,[1] Mateusz Marianski,[3,4,*] and Kevin Pagel[1,2,*]

[1]*Institute of Chemistry and Biochemistry, Freie Universität Berlin, Altensteinstraße 23a, 14195 Berlin, Germany.*

[2]*Department of Molecular Physics, Fritz-Haber-Institut der Max-Planck-Gesellschaft, Faradayweg 4–6, 14195 Berlin, Germany.*

[3]*Department of Chemistry, Hunter College, The City University of New York, New York, NY 10065, USA*

[4]*PhD Programs in Chemistry and Biochemistry, The Graduate Center, The City University of New York, New York, NY 10028, USA*

# Contributed equally

* Corresponding address: Prof. Mateusz Marianski: mmarians@hunter.cuny.edu; Prof. Kevin Pagel: kevin.pagel@fu-berlin.de

Table of Contents

Figures:

Figure S1 – IM-MS analysis of the used standard. p.3

Figure S2 – Relative energies of four labelled isomers p.4

Figure S3 – Trajectories and normalized structural distribution histograms of four labelled isomers p.5

Figure S4 – Conformations of GlcNAc-4S-ProA and GlcNAc-5S-ProA p.6

Figure S5 – Survival yield curves of GlcNAc6S-ProA and GlcNAc3S-ProA p.7

Figure S6 – TW-IMS ATD of HS2SNAc-ProA after activation at different CID voltages. p.7

Figure S7 – Relative migration from *6O*- to *3O*- during activation of the GlcNAc6S-ProA standard at different CID voltages. p.8

XYZ coordinates of the most stable conformers of GlcNAc-3S-ProA and GlcNAc-6S-ProA p.9

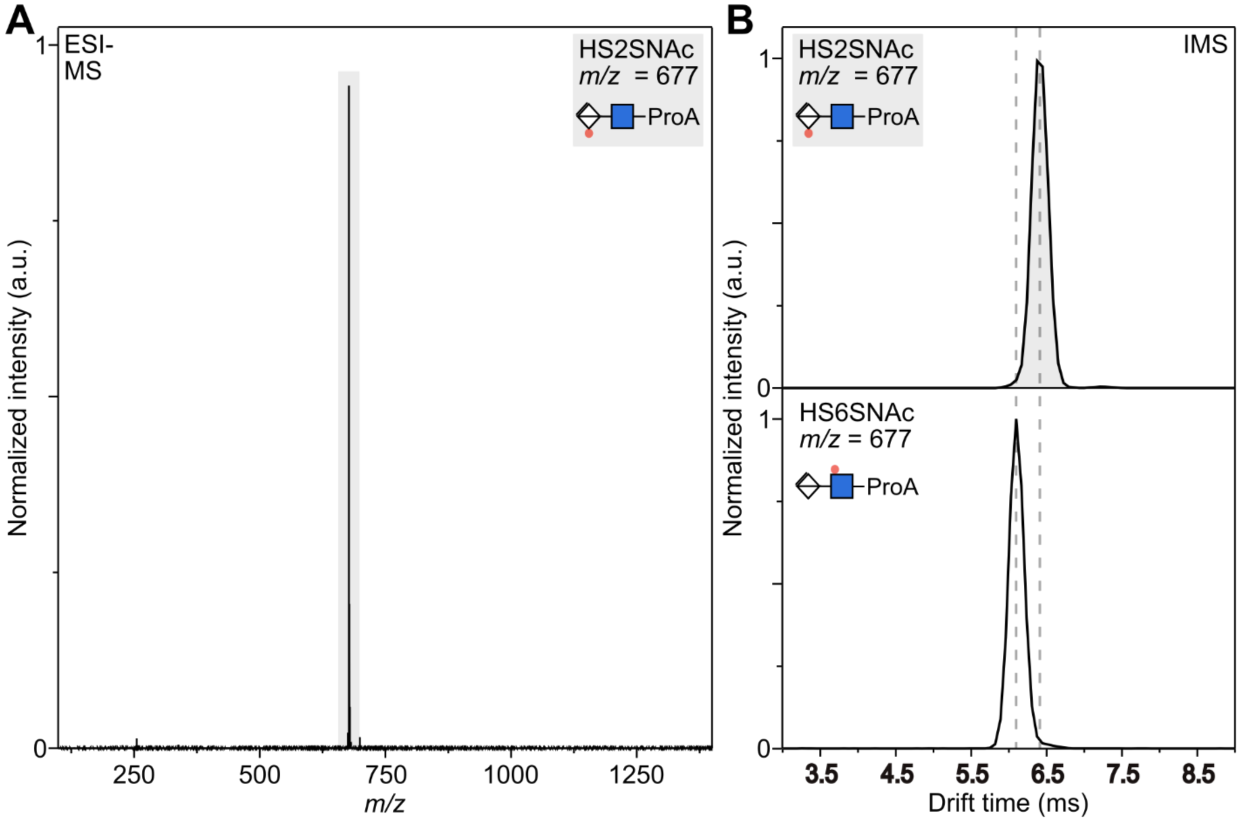

Figure S1: IM-MS analysis of the used standard. a) The mass spectrum of the used HS2SNAc-ProA shows no major contamination. b) IMS arrival time distributions of HS2SNAc-ProA and HS6SNAc-ProA. No migration of HS2SNAc-ProA prior to CID is visible.

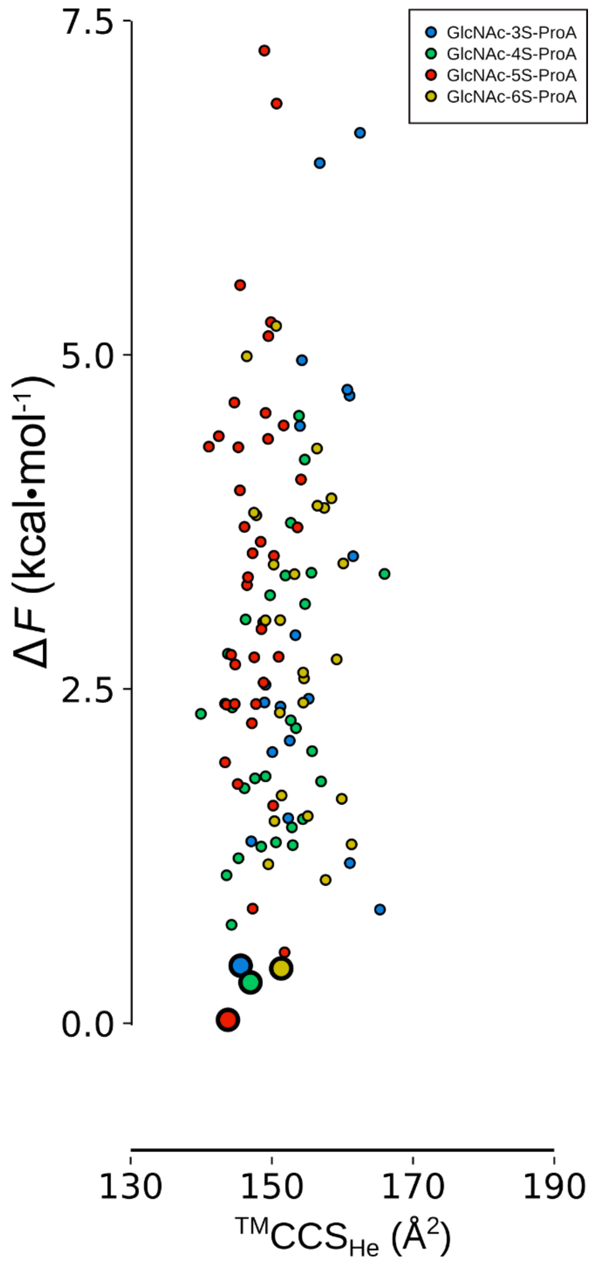

Figure S2: Relative energies of four labelled isomers against $^{TM}CCS_{He}$ plot found using CREST conformational sampling tool. Larger circles represent the most stable conformers of four different isomers. The global minimum, GlcNAc-5S-ProA, is about 0.5 kcal mol^{-1} more stable than the others.

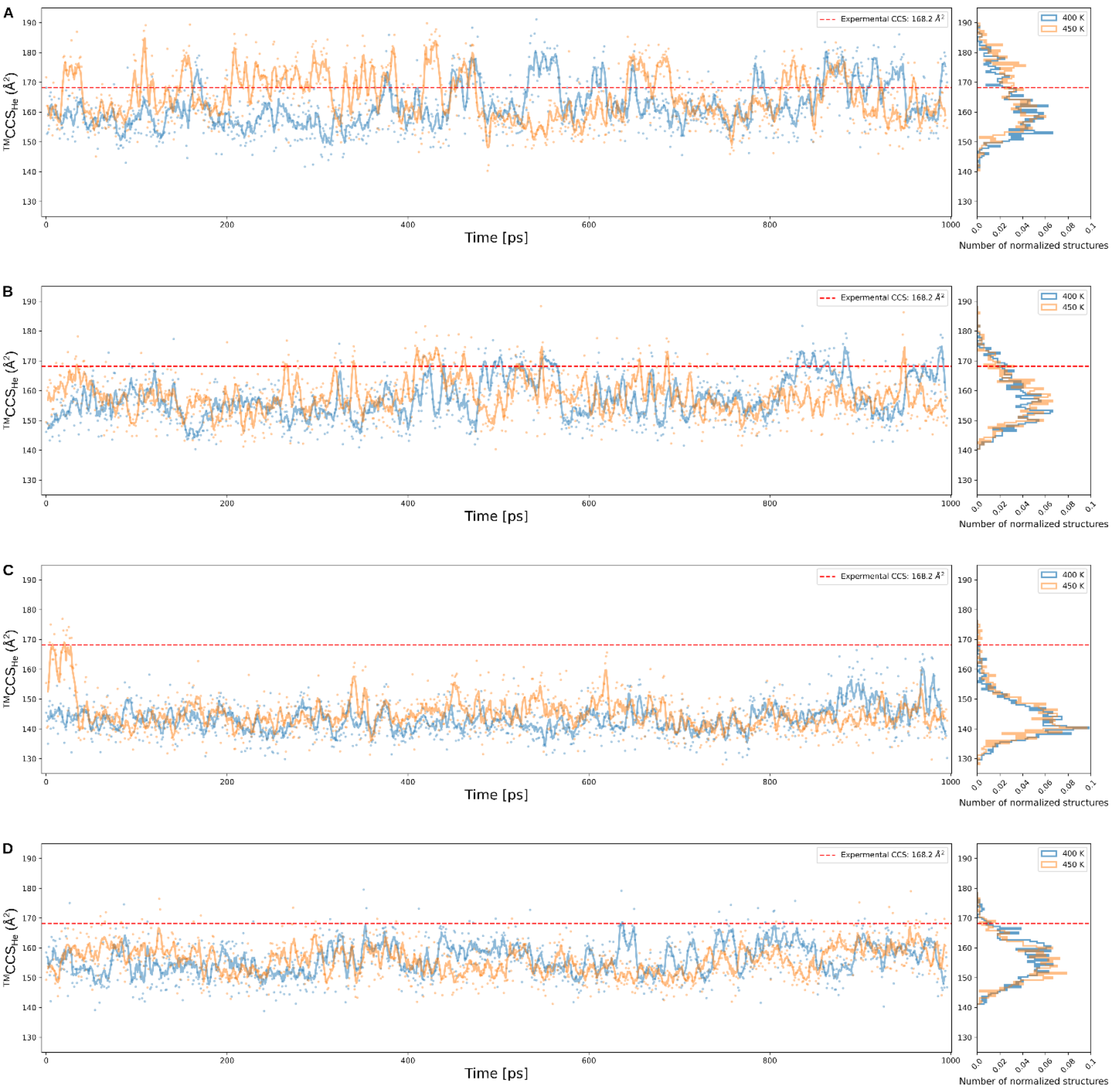

Figure S3: Collision cross-section values trajectories and normalized structural distribution histograms of GlcNAc-3S-proA, GlcNAc-4S-proA, GlcNAc-5S-proA, and GlcNAc-6S-proA, respectively. Each trajectory shows $^{TM}CCS_{He}$ calculations which were performed on 1 ns xTB trajectories at 400 and 450 K.

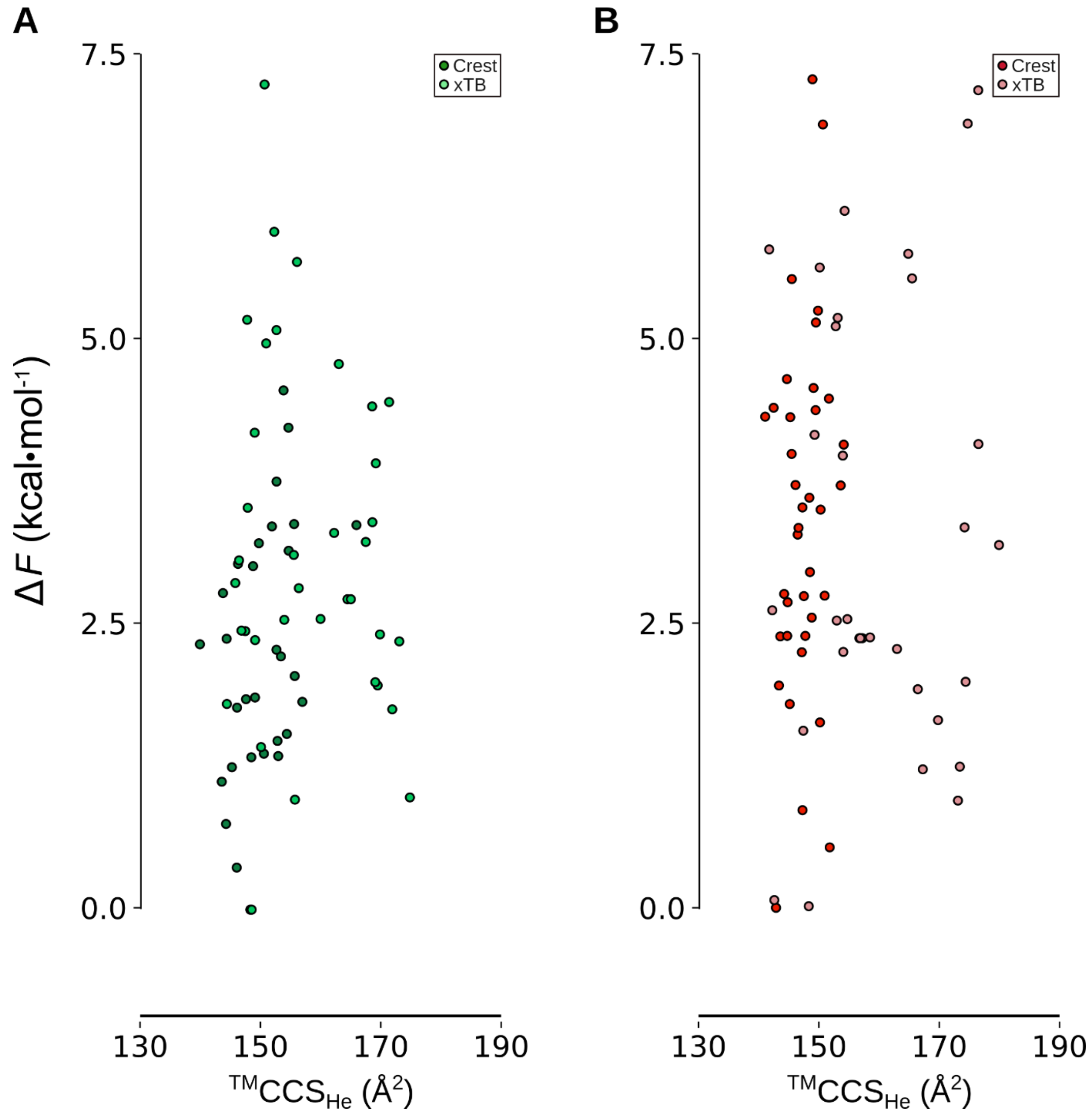

Figure S4: Thermodynamic stability of A) GlcNAc-4S-ProA (green dots), and B) GlcNAc-5S-ProA (red dots) conformers against their simulated collision cross-section values plots. The conformational sampling emerged via CREST and xTB methods and thermodynamic properties were calculated using DFT.

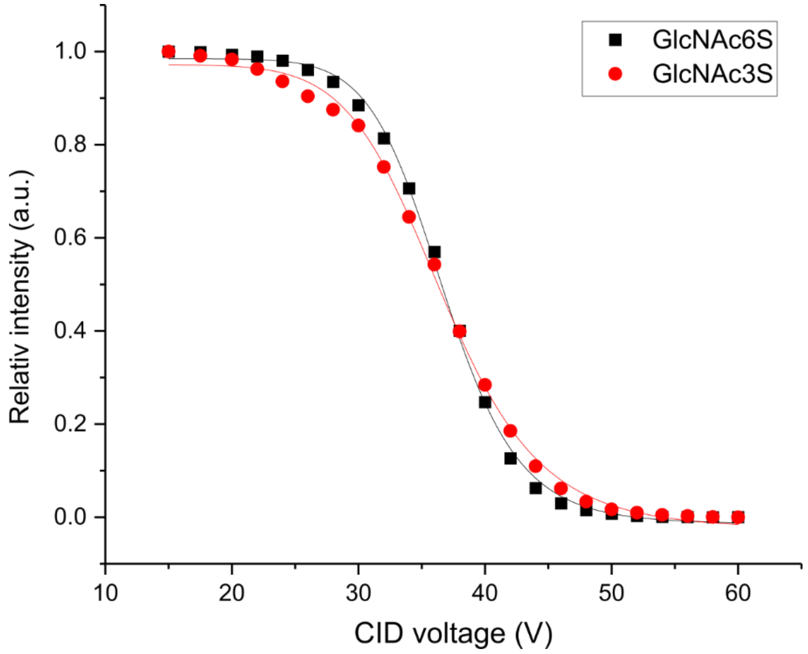

Figure S5: Survival yield curves of GlcNAc6S-ProA (black) and GlcNAc3S-ProA (red) fitted with sigmoidal functions.

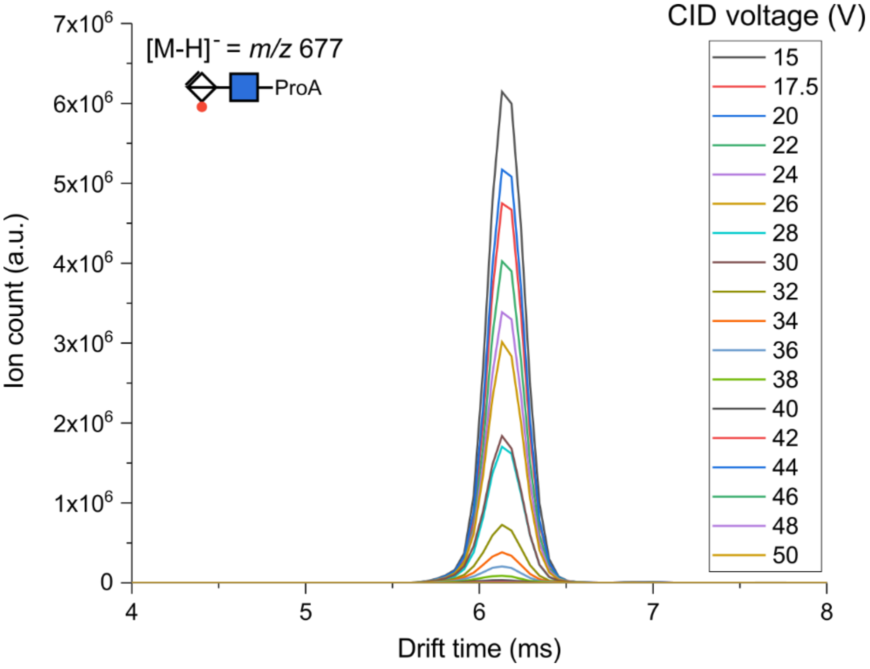

Figure S6: TW-IMS ATD of HS2SNAc-ProA after activation at different CID voltages. No change in drift time is observed.

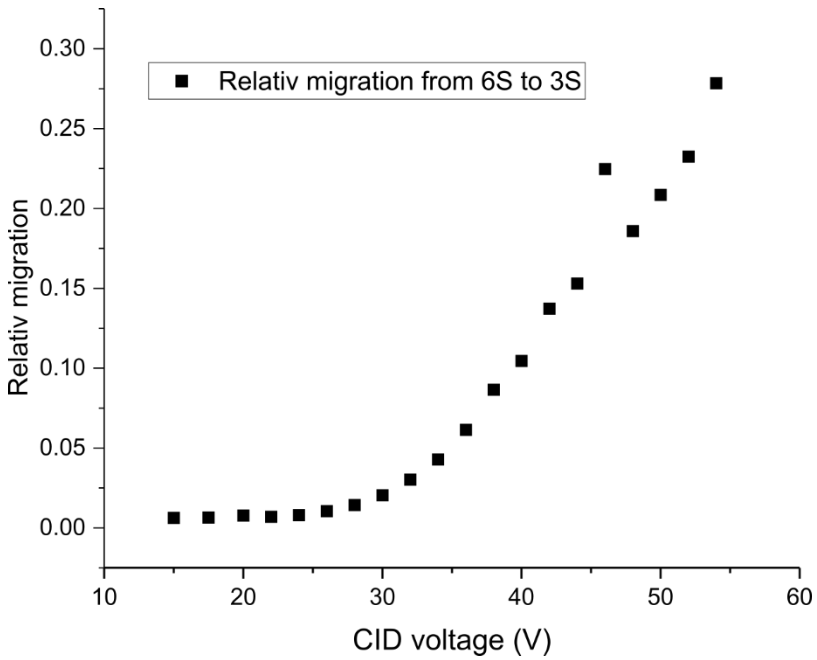

Figure S7: Relative migration from *6O*- to *3O*- during activation of the GlcNAc6S-ProA standard at different CID voltages.

XYZ structure of the most stable conformers of GlcNAc-3S-ProA

C	-2.514302	2.294254	-0.552120
C	-3.558063	1.358418	-1.134841
N	-4.897237	1.558317	-0.582363
C	-5.495180	2.821352	-0.969824
C	-6.653792	3.223930	-0.073001
C	-5.782110	0.414377	-0.795955
C	-6.118566	-0.276541	0.525346
N	-4.902326	-0.592763	1.240525
C	-4.231441	-1.754584	0.948258
O	-4.808223	-2.716514	0.455201
C	-2.773981	-1.759489	1.219374
C	-2.070688	-0.687755	1.784466
C	-0.694523	-0.692787	1.859852
C	0.050588	-1.779885	1.366890
N	1.422805	-1.729437	1.448290
C	2.286767	-2.326121	0.449813
C	2.490732	-1.460703	-0.809732
N	1.223501	-1.086087	-1.395695
C	0.456387	-1.965232	-2.072168
C	-0.885826	-1.431405	-2.508698
O	0.804256	-3.118207	-2.298380
C	3.321208	-0.199199	-0.574929
O	2.842245	0.512642	0.575908
S	2.037941	1.970259	0.288145
O	1.733918	2.376452	1.648547
O	3.035752	2.778699	-0.430163
O	0.886342	1.593810	-0.544324
C	4.812651	-0.491505	-0.401148
O	5.292644	-1.047125	-1.614343
C	5.674974	0.732273	-0.066444
O	5.563747	1.701260	-1.091975
C	5.473081	1.361959	1.320370
O	5.087055	0.437711	2.315370
C	-0.652085	-2.879771	0.846220
C	-2.031464	-2.856685	0.774096
H	-1.506258	1.988610	-0.842078
H	-2.647888	3.330161	-0.876417
H	-2.555289	2.275432	0.540791
H	-3.567403	1.437886	-2.239491
H	-3.265361	0.330312	-0.904325
H	-4.723545	3.592369	-0.909418
H	-5.831191	2.796284	-2.026195

H	-7.055200	4.194928	-0.379134
H	-7.473594	2.500574	-0.114967
H	-6.320998	3.296110	0.966210
H	-6.706692	0.725444	-1.300771
H	-5.298514	-0.316155	-1.456118
H	-6.732988	0.380997	1.148353
H	-6.662156	-1.206823	0.350630
H	-4.330448	0.225200	1.391948
H	-2.587418	0.187605	2.166317
H	-0.169522	0.174058	2.251273
H	1.751658	-0.810125	1.709418
H	3.257665	-2.540057	0.908313
H	1.873860	-3.282023	0.127571
H	3.036726	-2.069890	-1.537472
H	0.865494	-0.158833	-1.173751
H	-1.112338	-1.810735	-3.506058
H	-1.642336	-1.814520	-1.817415
H	-0.928465	-0.340150	-2.503524
H	3.215700	0.444394	-1.456962
H	4.967379	-1.244567	0.376372
H	5.440630	-0.291387	-2.196584
H	6.701472	0.353328	-0.135753
H	4.724360	2.178734	-0.953989
H	6.421844	1.800444	1.642657
H	4.748564	2.177460	1.239165
H	4.137398	0.326147	2.194685
H	-0.116190	-3.735099	0.452792
H	-2.571288	-3.689696	0.336623

XYZ structure of the most stable conformers of GlcNAc-6S-ProA

C	-4.141319	-3.079763	1.478144
C	-4.395286	-2.000676	0.437728
N	-4.447006	-0.655598	0.991473
C	-3.184845	-0.231288	1.590676
C	-3.274417	1.107807	2.302384
C	-5.082764	0.288747	0.092050
C	-4.530822	0.423800	-1.335244
N	-3.150856	0.838761	-1.414733
C	-2.783180	2.118735	-1.173090
O	-3.598297	3.007471	-0.930545
C	-1.317172	2.367591	-1.183410
C	-0.809993	3.432829	-0.437376

C	0.552007	3.626170	-0.306235
C	1.465097	2.757532	-0.927544
N	2.825823	2.930606	-0.720221
C	3.708020	1.787016	-0.784389
C	3.699388	0.897371	0.483847
N	2.408535	0.834043	1.132551
C	2.013878	1.765845	2.031286
C	0.627613	1.560605	2.584631
O	2.727421	2.700644	2.381651
C	4.235129	-0.502449	0.154091
O	4.716932	-1.156948	1.302972
S	-0.424148	-2.104619	-0.512347
O	0.191933	-1.174943	0.473799
O	-1.287447	-1.439911	-1.483869
O	-0.938629	-3.323554	0.095271
C	3.206736	-1.319904	-0.662804
O	3.723086	-1.513299	-1.976373
C	2.843800	-2.661433	-0.015884
O	2.400950	-2.470784	1.317754
C	1.830047	-3.421444	-0.866765
O	0.847439	-2.578985	-1.473803
C	0.951769	1.747725	-1.752338
C	-0.412900	1.552283	-1.861970
H	-3.105539	-3.077043	1.823016
H	-4.809981	-2.952280	2.335739
H	-4.319386	-4.064705	1.036378
H	-3.637183	-2.097413	-0.357726
H	-5.371217	-2.183179	-0.031083
H	-2.914169	-0.993042	2.327472
H	-2.356813	-0.206305	0.867478
H	-3.377984	1.946031	1.608721
H	-4.125736	1.120655	2.991002
H	-2.361660	1.275098	2.880635
H	-6.138058	-0.006183	-0.005613
H	-5.077702	1.277821	0.552713
H	-4.616303	-0.526317	-1.870015
H	-5.146918	1.161177	-1.861060
H	-2.443084	0.110570	-1.426783
H	-1.513606	4.086096	0.068376
H	0.932200	4.424671	0.324953
H	3.007178	3.493032	0.102197
H	3.466348	1.195380	-1.668845
H	4.732553	2.141898	-0.936692
H	4.375272	1.343135	1.217756

H	1.689329	0.212297	0.778083
H	0.071182	0.786565	2.053268
H	0.713323	1.285564	3.639777
H	0.086414	2.505863	2.524110
H	5.103246	-0.370627	-0.500911
H	2.274710	-0.754156	-0.752312
H	2.967978	-1.703052	-2.541062
H	3.748852	-3.273976	0.050718
H	1.537703	-2.013647	1.272394
H	2.336276	-3.903417	-1.706491
H	1.341304	-4.190738	-0.260564
H	3.941071	-1.521249	1.758158
H	1.615049	1.081769	-2.290497
H	-0.765574	0.728806	-2.470794
